# Digital health technologies and adherence to tuberculosis treatment

**DOI:** 10.2471/BLT.21.286021

**Published:** 2021-05-01

**Authors:** Hussain Abbas Zaidi, Charles D Wells

**Affiliations:** a Swarthmore College, Lang Center for Social and Civic Responsibility, 500 College Avenue, Swarthmore, PA 19081, United States of America (USA).; b Bill & Melinda Gates Medical Research Institute, Cambridge, USA.

The World Health Organization (WHO) estimates that one fourth of the world’s population has *Mycobacterium tuberculosis* infection and is at risk of developing active tuberculosis.^1^ The current standard treatment for drug-sensitive tuberculosis is a multidrug regimen consisting of rifampicin, isoniazid, pyrazinamide and ethambutol, administered for a minimum of 6 months.^2^ Though highly efficacious, the duration and complexity of this regimen may result in nonadherence, leading to suboptimal response (failure and relapse), emergence of drug resistance and continuous spread of the disease.^2^

To meet the tuberculosis-related sustainable development goals and the *End TB strategy* global control targets, novel regimens that can overcome deficiencies of existing treatments are needed to accelerate the decline in global tuberculosis incidence.^3^ In the four decades since the current 6-month regimen was established as standard treatment, at least three large international multicentre phase 3 trials demonstrated that four different 4-month regimens did not perform as well as the 6-month regimen.^4^^–^^6^ With an expanded pipeline of potential new tuberculosis agents to bring into combination development, many innovative development strategies have evolved and are being implemented to accelerate development of shorter and simpler tuberculosis treatment regimens.

With these innovative clinical trials to evaluate potential tuberculosis regimens, patient adherence and retention in trials is critical to establish robust efficacy and safety profiles for investigational regimens. In the phase 2 trial that supported initial registration of bedaquiline with the United States of America Food and Drug Administration in 2012, patient adherence and retention were problematic. The drug was approved but received a black box warning – that is, the highest warning for drugs and medical devices – due to a death imbalance between the bedaquiline-containing experimental arm (10 deaths) versus the control arm (2 deaths).^7^

WHO’s recommendation to intensify the use of digital health technologies to contain the coronavirus disease 2019 (COVID-19) pandemic, has potential as a strategy to promote adherence and retention to tuberculosis treatment during clinical trials and in routine care.^8^ In electronic dose monitoring systems, such as medication event reminder monitoring systems, patient medication is dispensed in containers with special lids that show whether the patient opened the box, serving as an indicator for medication ingestion. A monitor helps patients organize medications and understand dosing, and reminds the patient to take or refill medication when needed. Similarly, 99DOTS is a low-cost, mobile phone-based digital health technology that can also allow real-time remote monitoring of medication intake.^9^ An antituberculosis treatment blister pack is wrapped in a custom envelope, with hidden phone numbers only revealed when a dose is dispensed, and then provided to the patient. After taking their medication, patients make a call to the phone number hidden until dose completion, allowing providers to ensure that the dose was in hand. The use of these event reminder monitoring systems and 99DOTS technology in tuberculosis regimen trials could serve to limit the number of patient visits to trial sites while still allowing for close monitoring of patient adherence and efforts to support patients for whom adherence is problematic.

Directly observed therapy by video recording serves as another viable clinical monitoring tool in the context of COVID-19. This protocol was developed as a flexible, low-burden method of providing remote directly observed therapy via smartphones, and involves patients video-recording themselves taking their medications or conducting a live-video call with an assigned health-care worker, and transferring the videos using a secure interface to workers for review. Studies have demonstrated the feasibility of this approach for monitoring medication ingestion among tuberculosis patients with a high fraction of expected doses observed, all while minimizing staff time and travel, an important feature to mitigate risk of contracting COVID-19.^10^^,^^11^

With robust evidence from their effective use in the context of tuberculosis control programmes, these tools can be used in tuberculosis clinical trials to support and monitor patients throughout the course of trial participation and achieve high levels of retention. Ensuring the successful execution of innovative trials designed to identify potentially transformative new tuberculosis regimens will contribute to the global effort to accelerate the decline and eventual elimination of this disease.
